# Room to Move: Enhancing Head and Neck Radiotherapy With a Six-Degree-of-Freedom Couch

**DOI:** 10.7759/cureus.102140

**Published:** 2026-01-23

**Authors:** Peter L Lee, Joseph V Panetta, Alexander P Lukez, Mihir K Karande, Thomas J Galloway

**Affiliations:** 1 Department of Radiation Oncology, Mass General Brigham, Boston, USA; 2 Department of Radiation Oncology, Fox Chase Cancer Center, Philadelphia, USA; 3 Department of Radiation Oncology, University of Vermont Medical Center, Burlington, USA

**Keywords:** head and neck oncology, head and neck radiation, image guided radiotherapy, immobilization, radiation simulation

## Abstract

Purpose

Image-guided radiation therapy (IGRT) allows for a reduction in target volume margins, but relies on accurate daily set-up. Six-degree-of-freedom couches offer rotational adjustments during alignment. Given the often large and irregular target volumes treated in head and neck (H&N) cancers, rotational errors may contribute significantly to inter-fraction variability in both target coverage and critical-structure dose. We seek to evaluate the impact of rotational adjustments on target coverage and critical-structure dose in H&N radiotherapy.

Methods

We reviewed 214 radiotherapy fractions for H&N cancer. Patients were treated using a six-degree-of-freedom couch that allows a maximum of ±3° of roll, pitch, and yaw, in addition to linear shifts. Daily couch adjustments were recorded, and the CT simulation images and treatment plan were imported into the oncology management system to allow fusion. The delivered dose was recalculated with and without rotational components, with deviation thresholds for planning target volume (PTV) V70Gy coverage taken to be 3% and 5%.

Results

A rotational shift in at least one plane was used on 212 of 214 treatment fractions. Without rotational shifts, PTV V100% was lower by a median of 1.97% (1.77%-2.18%; 95% CI) compared to the planned coverage. Specifically, 43 (20%) fractions had a decrease in PTV V100% of ≥3% when rotational shifts were not applied, and 10 (4.7%) fractions had a decrease in PTV V100% of ≥5%. All 43 fractions with a ≥3% drop in PTV coverage required at least 1.5° rotational correction in at least one direction. Organs at risk (OAR) dose was less dependent on rotational correction, without significant correlations between the magnitude of rotational correction and increased OAR dose.

Conclusion

Rotational corrections are applied frequently when treating H&N cancers on a six-degree-of-freedom couch. Rotational adjustments had the greatest benefit on PTV coverage, while OAR doses were less dependent. These data highlight the utility of a six-degree-of-freedom couch for H&N radiotherapy and suggest a target set-up goal of less than 1.5° rotational error in all planes.

## Introduction

Modern radiotherapy has evolved to allow for high doses of radiation to be delivered to complex target volumes while sparing nearby critical structures. Various technological advancements have been introduced to allow for increased accuracy and precision of radiation delivery, including modalities such as daily image guidance, surface-based or X-ray-based tracking, non-gantry-based accelerators, and improved patient immobilization devices. Specifically, in head and neck (H&N) cancers, there is increasing utilization of intensity-modulated radiation therapy (IMRT), with evidence supporting benefits in toxicity without compromise of loco-regional control compared to traditional two-dimensional radiation therapy (2D-RT) or three-dimensional conformal radiation therapy (3D-CRT) [1,2]. Given that IMRT often involves smaller treatment margins compared to 3D and 2D techniques, accurate and precise daily patient set-up is critical to ensure proper delivery of RT.

Typical set-up techniques for H&N radiotherapy include a thermoplastic mask for immobilization, isocenter marking, and image guidance with remote shifts of the treatment couch to align based on anatomy visualized - most commonly by cone beam CT (CBCT) - though other imaging modalities may be employed as well, including kV or MV X-ray imaging [3,4]. Further advancements include customizable intra-oral devices to both immobilize and displace non-involved tissue [5], real-time 3D-printed bolus when covering superficial targets [6,7], and improvements in patient set-up accuracy and precision. One such device that has been implemented in some clinics is the use of a six-degree-of-freedom treatment couch. This device, in conjunction with image guidance, allows a patient to be rotated about the pitch, yaw, and roll axes, along with traditional 3D translations [8]. A prior study has reported that the use of a six-degree-of-freedom couch for H&N cancers resulted in better intrafraction reproducibility [9]; however, these advanced treatment couches are not universally available, particularly in resource-limited settings. With the ability to make and record rotational adjustments, we now seek to better understand the dosimetric impact of rotational errors and identify potential alignment goals to optimize patient set-up.

In this study, we retrospectively reviewed 214 total treatment fractions from six individual courses of definitive IMRT for H&N cancers. Daily CBCT images for each fraction were co-registered to the planning CT with and without rotational corrections, allowing for dosimetric comparison between plans.

## Materials and methods

Patient selection and radiation technique

For this analysis, 214 treatment fractions were retrospectively collected from six individual treatment courses of definitive radiotherapy for H&N cancer. This number was selected to provide sufficient individual fractions for exploratory dosimetric analysis, with the understanding that there may be confounding factors attributed to patient-specific features. Inclusion criteria encompassed any patient planned for conventionally fractionated H&N radiotherapy. All patients were required to have a primary high-dose target volume, as well as a low-dose elective nodal volume. Multiple high-dose target volumes were allowed. Elective nodal volumes could be either bilateral or unilateral in target coverage. The planning target volume (PTV) was defined as the clinical target volume with a uniform 3-mm expansion. All courses were planned with volumetric modulated arc therapy (VMAT) to be delivered in daily fractions. Patients unable to tolerate standard immobilization techniques were excluded from this study, as were patients who required re-simulation and re-planning during their treatment course.

Simulation and immobilization

All patients were set up for simulation in the supine arms-down position. Legs and knees were supported with a foam cushion, allowing knee flexion for comfort. A five-point thermoplastic mask was made for each patient and secured to the treatment table, each marked with reference points to identify the treatment isocenter. CT simulation was performed using a Siemens Somatom Definition AS (Siemens Healthineers, Erlangen, Germany) scanner with 2-mm slice thickness.

Daily treatment

Patients were set up in simulation position, with initial manual alignment of the in-room lasers to the isocenter markings placed on the thermoplastic mask at the judgment of the radiation therapists. Mask fit was assessed daily by the treatment team. If a change in fit was significant enough to require re-simulation and re-planning, these patients were excluded from this study. A daily CBCT was then obtained and coregistered to the planning CT. From there, additional optimization of alignment, most often to bony anatomy, was done by the radiation treatment team for both translations and rotations. Couch corrections were provided by the remote automatic table movement device and the six-degrees-of-freedom treatment couch. A maximum of 3° of rotation on any axis, in 0.1° increments, is allowed by the treatment couch. When greater than 3° of rotation was required to align the CBCT with the planning CT, the patient was manually adjusted, and a new CBCT was obtained. All daily shifts and rotations were recorded in the oncology information system.

Review of daily shifts and dosimetry

**Figure 1:**
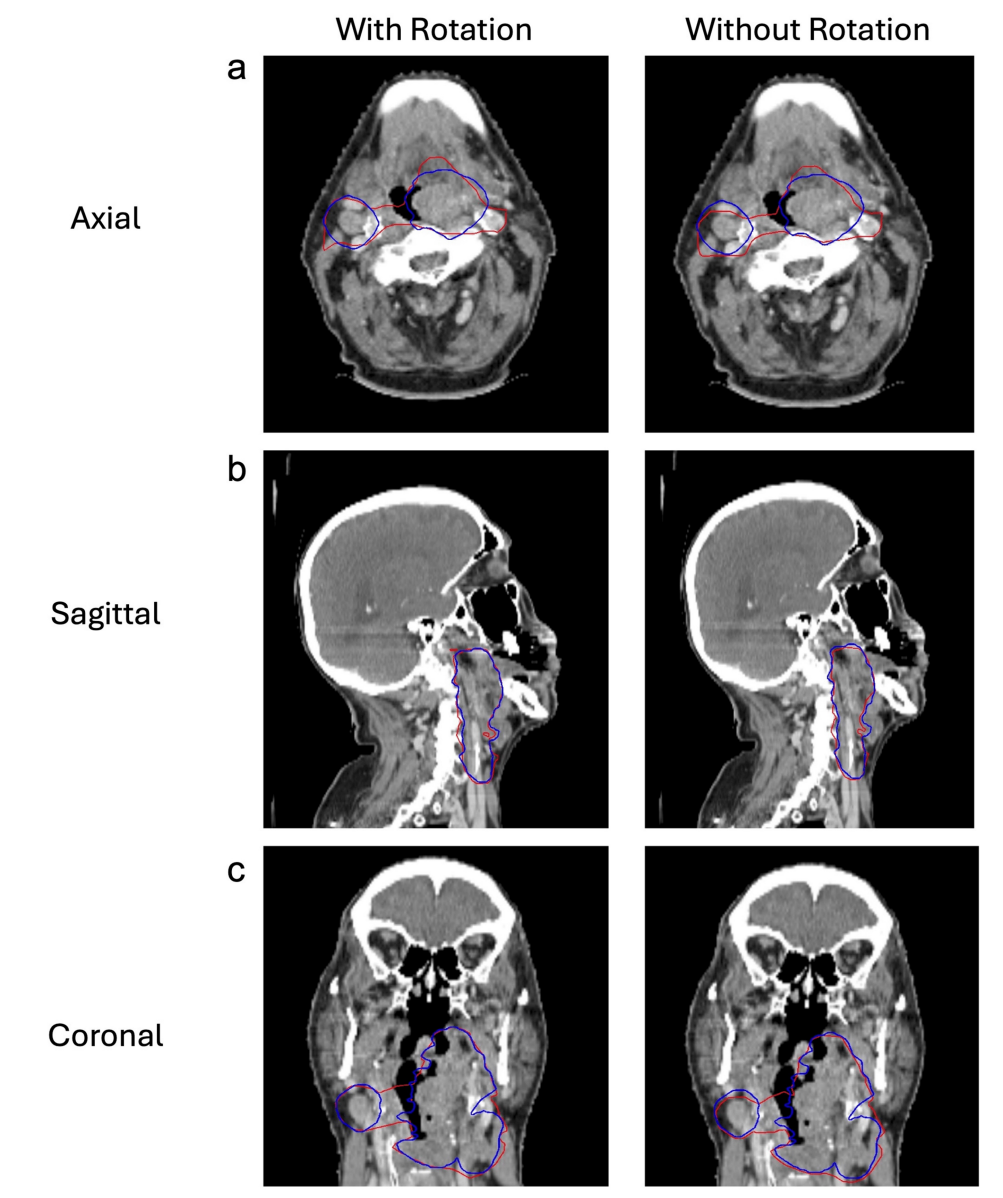
Treatment Plan With and Without Daily Rotational Correction Representative images of the treatment plan with and without daily rotational correction. Red outline: 100% isodose line and blue outline: planning target volume (PTV). Shown on a) axial, b) sagittal, and c) coronal planes.

Daily shifts, as recorded above, were extracted and imported into a treatment analysis software (Varian Velocity; Varian Medical Systems, Palo Alto, CA, USA), allowing for dosimetric analysis of target volumes and organs at risk (OARs). Linear shifts were recorded in the axial, sagittal, and coronal axes in 1-mm increments, and rotations of roll, pitch, and yaw in 0.1° increments. Rotational corrections were then removed for each fraction, allowing for review of the treatment plan and comparison of dose-volume histograms with and without rotational corrections. No additional translational adjustments were made after the rotational corrections were removed. Translations were recorded as a root-sum-square value to give the total linear displacement of the isocenter. Rotations were recorded both on individual axes as well as a total sum of rotational changes (Figure 1).

Statistics

Mean, median, SD, SEM, and 95% CI of translations and rotations were calculated. Dosimetric goals, including target coverage and OAR dose constraints, were plotted against maximum rotational adjustments, with best linear fits applied as appropriate. Deviation thresholds for PTV V70Gy coverage were 3% and 5%, in line with daily LINAC dosimetric QA recommendations per AAPM TG-142. Statistical analysis and linear fits were performed using GraphPad Prism 10, version 10.5.0 (673) software (GraphPad Software, San Diego, CA, USA).

## Results

A rotational adjustment of nonzero magnitude was required on 212 of 214 analyzed treatment fractions. Collectively, 101 (47%), 77 (36%), and 34 (16%) fractions required rotations of 2.1-3°, 1.1-2°, and 0.1-1°, respectively, about at least one individual axis, with two fractions requiring no rotational adjustment. The median magnitude of roll, pitch, and yaw adjustment was 1.3° (1-1.5°; 95% CI), 0.9° (0.8-1.1°; 95% CI), and 0.8° (0.7-0.9°; 95% CI), respectively. For roll, six fractions (3%) did not require any adjustment, 89 fractions (42%) were rotated by a magnitude of 0.1-1°, 55 fractions (26%) by 1.1-2°, and 64 fractions (30%) by 2.1-3°. These figures were 6 (3%), 113 (53%), 62 (29%), and 33 (15%) for pitch, and 8 (4%), 122 (57%), 60 (28%), and 24 (11%) for yaw (Table 1).

**Table 1:** Frequency and Magnitude of Rotational Corrections Along Each Axis

Axis of Rotation	Total Fractions (n)	No Rotation (n, %)	0.1-1° (n, %)	1.1-2° (n, %)	2.1-3° (n, %)	Median Rotation (Degrees)
Any Axis	214	2 (1%)	34 (16%)	77 (36%)	101 (47%)	-
Pitch	214	6 (3%)	89 (46%)	55 (26%)	64 (30%)	1.3°
Yaw	214	6 (3%)	113 (53%)	62 (29%)	33 (15%)	0.9°
Roll	214	8 (4%)	122 (57%)	60 (28%)	24 (11%)	0.8°

When using 1.5° as an inflection point (50% of the maximum rotational capability of the treatment couch), we find that 86 (40%) fractions required greater than 1.5° of roll adjustment, 52 (24%) fractions required greater than 1.5° of pitch adjustment, and 45 (21%) fractions required greater than 1.5° of yaw adjustment.

**Figure 2:**
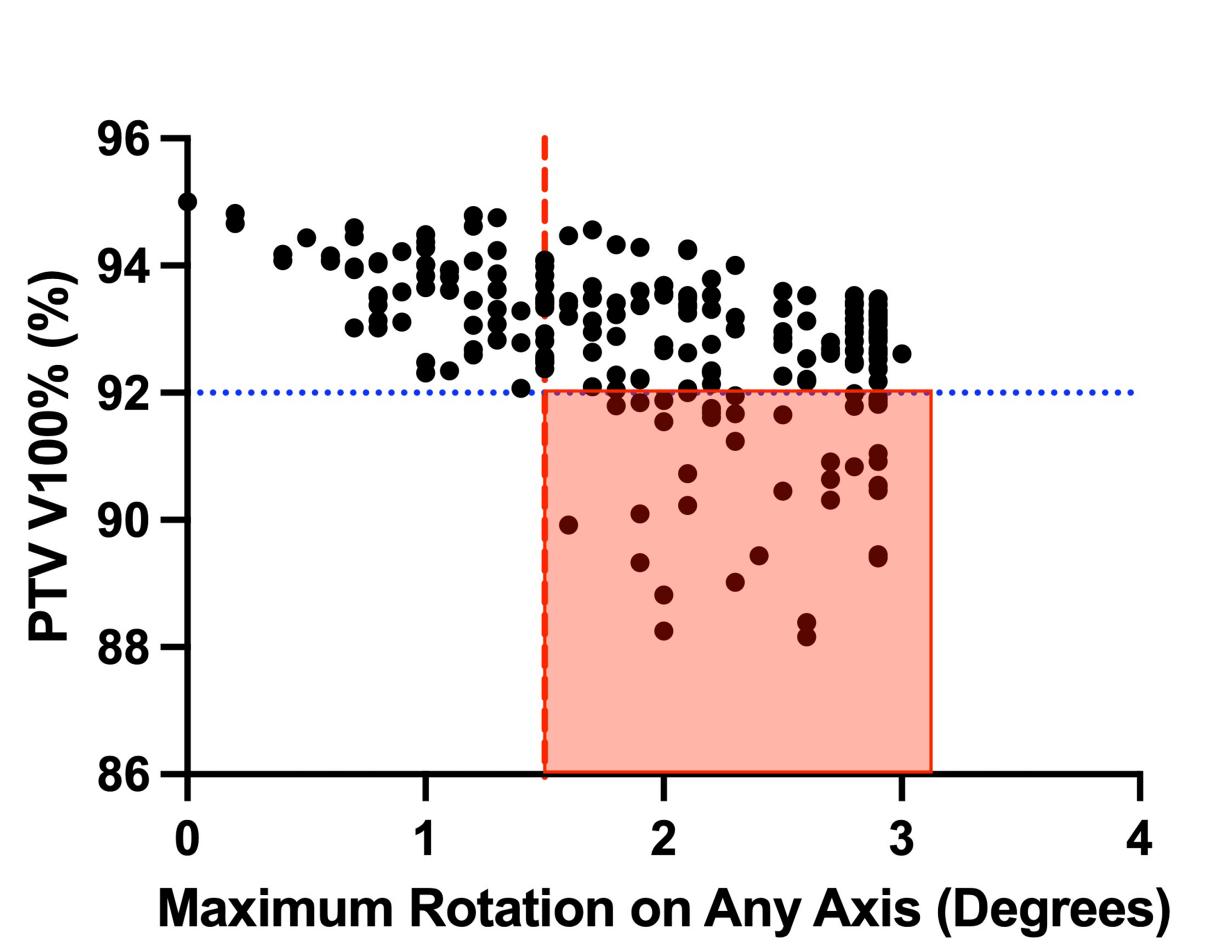
PTV V100% Coverage V100% coverage of the planning target volume (PTV) per fraction, without rotational adjustment, plotted against the maximum degree of rotation about any individual axis. The red area indicates fractions that would have PTV coverage of 92% or less if rotations were not applied.

Of the 214 fractions, 43 fractions had a decrease in PTV V100% of 3% or greater when rotational adjustments were not used, and 10 fractions had a decrease in PTV V100% of 5% or greater. Notably, all 43 fractions with a greater than 3% drop in PTV coverage required at least a 1.5° rotational adjustment in any direction. Of fractions where the degree of rotation was kept at 1.5° or less in all planes (68 fractions total), PTV coverage never decreased by greater than 3% (2.69% maximum) (Figure 2).

In the absence of rotational adjustment, PTV V100% coverage was found to be lower by a median of 1.97% (1.77%-2.18%; 95% CI). When plotting the largest degree of rotational adjustment on any plane against PTV coverage, we find a linear fit slope of -0.84, with an R² of 0.25 (p < 0.0001).

**Figure 3:**
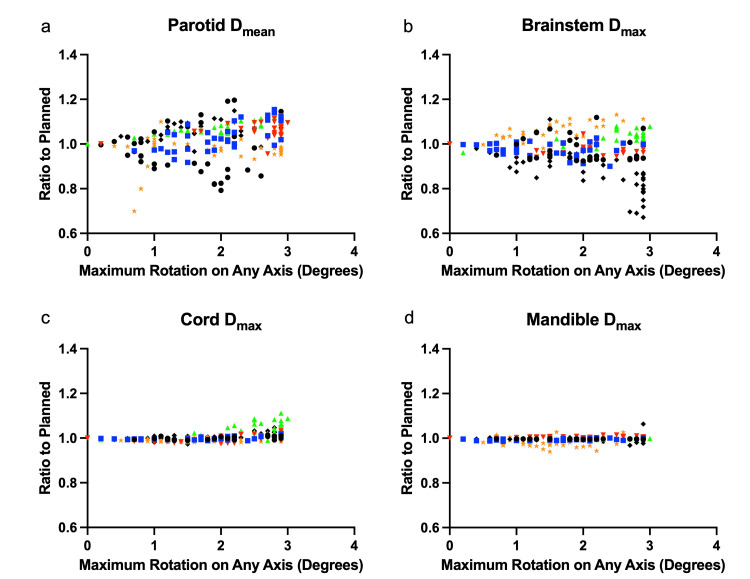
Dose to Organs at Risk Doses to respective organs at risk (OARs) without rotational adjustment, relative to the planned dose per fraction, plotted against the maximum degree of rotation about any axis. Each unique symbol corresponds to an individual patient treatment course (6 patients total). a) Parotid mean dose (Dmean); b) Brainstem maximum dose (Dmax); c) Spinal cord Dmax; d) Mandible Dmax.

Regarding OARs, the contralateral parotid mean dose exceeded 105% of the planned dose in 96 (45%) fractions, and exceeded 110% of the planned dose in 25 (12%) fractions when rotational adjustments were omitted. In one patient, the absence of rotational adjustment would have led to a total contralateral parotid mean dose of 107% of the planned, translating into a 21.43 Gy mean dose, exceeding our constraint of 20 Gy (Figure 3a). For the brainstem, 34 fractions would have received >105% of the planned dose, and 10 fractions >110% of the planned dose. Cord Dmax exceeded 105% of the planned dose in 14 fractions, and 110% in one fraction. Mandible Dmax exceeded 105% of the planned dose in 1 fraction, with no fractions exceeding 110%. We did not find any patients in whom the absence of rotational correction would have led to overall treatment exceeding the critical dose constraints of the spinal cord and brainstem (Figures 3b-3d).

## Discussion

Utilization of six-degree-of-freedom

In this study, we find that utilization of rotational adjustments with a six-degree-of-freedom treatment couch was nearly universal, with only 2 of 214 total fractions where rotations were not needed to optimize daily alignment. The necessity of rotational adjustment may be related to several factors, including movement of the head within the immobilization mask, limitations of external marking precision, and daily subjectivity in initial patient positioning. These data support prior studies that have demonstrated the utility of six-degree-of-freedom couches [10,11]. This study also shows that, despite using individual head support systems with thermoplastic mask immobilization and isocenter markings, additional image-guided remote rotational corrections further aid in daily alignment, supporting prior findings [12,13]. When analyzed on a per-axis basis, we found that pitch more often required large adjustments (greater than 1.5°, or 50% of table capacity), occurring 40% of the time, compared to 24% for roll and only 22% for yaw. This may suggest that, in the absence of remote rotational couch technology, a greater emphasis on correct pitch when positioning patients may be beneficial.

(A) Immobilization masks always allow some movement of the head, since the thermoplastic masks don’t conform perfectly to the skin as they harden. While there are tighter and more reinforced masks available (e.g., masks designed for stereotactic radiosurgery (SRS)), these are less comfortable and not routinely used, since the larger margins involved with IMRT treatments make them less critical. (B) Even with masks that offer perfect immobilization, patients are set up to align markings in four dimensions: x, y, z, and yaw. The other rotations can’t be adjusted easily, since the therapists don’t rotate the couch on those axes manually while setting up - they are controlled remotely by the Hexapod after CBCT alignment. (C) Even if the therapists aligned along those axes, setup accuracy is reliant upon laser accuracy and the precision of the therapists’ setup on a daily basis.

Target volume coverage

Given that modern, inverse-planned radiation treatments are often optimized to a goal of PTV V100% coverage of 95% [14], we were interested in better understanding the impact of rotational adjustments on PTV coverage. Of particular interest were thresholds of 3% deviation, which is in line with AAPM TG-142 guidelines for a daily QA output tolerance of ±3%. Another threshold of 5% may also be relevant based on the ICRU recommendation that the delivered dose remain within ±5% of the prescribed dose [15,16]. In the absence of rotational adjustments, we found that the median PTV V100% coverage was lower by 1.97%, within the 3% daily QA output tolerance. Generally, we observed a trend toward lower target coverage with greater maximum rotational corrections on any plane, with a linear regression suggesting a decrease in PTV V100% of 0.84% per 1° rotational change. More notably, 43 fractions (20%) saw a decrease in PTV V100% exceeding the 3% threshold, all of which required at least 1.5° of correction on any axis. Conversely, of the 68 fractions where rotational corrections remained less than 1.5° on any axis, none showed a PTV V100% reduction greater than 3%. These data suggest that a clinical alignment goal of less than 1.5° rotation along all axes, which can be measured by some image-guided systems even in the absence of a six-degree-of-freedom couch, may help optimize daily PTV coverage. It is important to note that, while these dosimetric measures are meaningful, this study does not explicitly evaluate clinical outcomes such as tumor control or toxicity.

Impact on OARs

Overall, we found a less significant correlation and impact of malrotation on dose to OAR. This is likely multifactorial, but may be in part attributed to typical relationships of isocenters to PTV versus OARs, the overall volume of PTV compared to OAR structures, and plan optimization goals that generally prioritize PTV coverage [17]. While a linear regression of cord Dmax did show a statistically significant deviation from zero, with a slope of 0.016 translating to a 1.6% increase in cord Dmax per degree of malrotation, this is likely not clinically meaningful. Our other OARs of interest, including parotid mean dose, brainstem max dose, and mandible max dose, did not show a linear regression with significant deviation from zero. In one patient whose initial plan had a parotid mean dose at our constraint level of 20 Gy, we observed that without rotational corrections, the parotid mean dose would have been 21.43 Gy, exceeding the 20 Gy dose constraint per QUANTEC [18]. In no cases would critical safety thresholds have been exceeded for brainstem or cord.

Limitations

There are several important limitations to consider in this study. First, the modeling of dose distribution is done in a retrospective manner by taking the aligned images and eliminating the rotational corrections. It is possible that, had rotations not been available to the treating team, the linear shifts applied would differ compared to what was used in conjunction with rotational correction. This method represents a worst-case scenario of daily rotational error for each analyzed fraction. We considered manually adjusting linear shifts after removal of rotations; however, we decided against this, given that it would rely on a subjective interpretation of optimal alignment and may introduce user bias. In an ideal setting, the utility of rotational corrections would be compared in real time against a three-degree-of-freedom couch; however, this may not necessarily be a reasonable use of resources in the clinical setting. The use of phantoms or virtual modeling may be an intriguing consideration for future studies. An additional limitation is the small number of patients (n = 6) in this study. Despite a large number of total treatment fractions allowing for statistical analysis, the limited number of individual patients contributing to these fractions may have introduced patient-specific variables that have not been accounted for. The single-institution nature of this study is also limiting, as alignment is often a subjective process, and alignment instructions and priorities may differ based on both clinical practice and institutional guidelines. Results from this study should be considered hypothesis-generating rather than definitive.

## Conclusions

This study demonstrates that, with modern daily CBCT-guided radiotherapy for H&N cancers, rotational misalignment along roll, pitch, and yaw is present in nearly all treatment fractions, with pitch being the most frequent. This did not result in clinically meaningful (>3%) deviation in PTV coverage for the majority of fractions; however, in the 20% of fractions where >3% deviation was present, all saw a rotational misalignment of at least 1.5° on any axis. OARs were not uniformly impacted by rotations. We recommend a clinical set-up goal to limit rotational misalignment to 1.5° or less when six-degree-of-freedom treatment couches are not available, with an emphasis on roll alignment.
